# Construction and Evaluation of Alginate Dialdehyde Grafted RGD Derivatives/Polyvinyl Alcohol/Cellulose Nanocrystals IPN Composite Hydrogels

**DOI:** 10.3390/molecules28186692

**Published:** 2023-09-19

**Authors:** Hongcai Wang, Ruhong Yin, Xiuqiong Chen, Ting Wu, Yanan Bu, Huiqiong Yan, Qiang Lin

**Affiliations:** 1 Key Laboratory of Water Pollution Treatment & Resource Reuse of Hainan Province, College of Chemistry and Chemical Engineering, Hainan Normal University, Haikou 571158, China; whcz0505@163.com (H.W.); chenxiuqiongedu@163.com (X.C.); wt09250916@163.com (T.W.); buynedu@163.com (Y.B.); linqianggroup@163.com (Q.L.); 2 Key Laboratory of Tropical Medicinal Resource Chemistry of Ministry of Education, College of Chemistry and Chemical Engineering, Hainan Normal University, Haikou 571158, China; 3 Key Laboratory of Natural Polymer Functional Material of Haikou City, College of Chemistry and Chemical Engineering, Hainan Normal University, Haikou 571158, China; 4 Hainan Hongta Cigarette Co., Ltd., Haikou 571100, China; hnhtyr@sina.com

**Keywords:** alginate, interpenetrating network composite hydrogels, cellular adhesion, cellular adhesion, tissue engineering

## Abstract

To enhance the mechanical strength and cell adhesion of alginate hydrogel, making it satisfy the requirements of an ideal tissue engineering scaffold, the grafting of Arg-Gly-Asp (RGD) polypeptide sequence onto the alginate molecular chain was conducted by oxidation of sodium periodate and subsequent reduction amination of 2-methylpyridine borane complex (2-PBC) to synthesize alginate dialdehyde grafted RGD derivatives (ADA-RGD) with good cellular affinity. The interpenetrating network (IPN) composite hydrogels of alginate/polyvinyl alcohol/cellulose nanocrystals (ALG/PVA/CNCs) were fabricated through a physical mixture of ion cross-linking of sodium alginate (SA) with hydroxyapatite/D-glucono-δ-lactone (HAP/GDL), and physical cross-linking of polyvinyl alcohol (PVA) by a freezing/thawing method, using cellulose nanocrystals (CNCs) as the reinforcement agent. The effects of the addition of CNCs and different contents of PVA on the morphology, thermal stability, mechanical properties, swelling, biodegradability, and cell compatibility of the IPN composite hydrogels were investigated, and the effect of RGD grafting on the biological properties of the IPN composite hydrogels was also studied. The resultant IPN ALG/PVA/CNCs composite hydrogels exhibited good pore structure and regular 3D morphology, whose pore size and porosity could be regulated by adjusting PVA content and the addition of CNCs. By increasing the PVA content, the number of physical cross-linking points in PVA increased, resulting in greater stress support for the IPN composite hydrogels of ALG/PVA/CNCs and consequently improving their mechanical characteristics. The creation of the IPN ALG/PVA/CNCs composite hydrogels’ physical cross-linking network through intramolecular or intermolecular hydrogen bonding led to improved thermal resistance and reduced swelling and biodegradation rate. Conversely, the ADA-RGD/PVA/CNCs IPN composite hydrogels exhibited a quicker degradation rate, attributed to the elimination of ADA-RGD by alkali. The results of the in vitro cytocompatibility showed that ALG/0.5PVA/0.3%CNCs and ADA-RGD/PVA/0.3%CNCs composite hydrogels showed better proliferative activity in comparison with other composite hydrogels, while ALG/PVA/0.3%CNCs and ADA-RGD/PVA/0.3%CNCs composite hydrogels displayed obvious proliferation effects, indicating that PVA, CNCs, and ADA-RGD with good biocompatibility were conducive to cell proliferation and differentiation for the IPN composite hydrogels.

## 1. Introduction

In recent years, tissue engineering scaffolds have been used for bone regeneration or repair because of their properties comparable to natural bone tissue [1,2]. From the point of view of material science, bone tissue scaffolds should be designed to mimic the structural characteristics of the extracellular matrix (ECM) of natural bone tissue [3]. The material should not only be conducive to cell adhesion, cell proliferation, and differentiation but also should be beneficial to the absorption of nutrients from the outside and the discharge of metabolic waste, as well as have a certain mechanical strength to meet the mechanical support required by the tissue in the process of repair [4,5,6]. On this basis, it is necessary to meet the characteristics of “tailor-made”, that is, to adjust according to the position and size of bone defects. Therefore, it is very important to select suitable bone tissue engineering materials and corresponding preparation methods.

Natural polymer materials, including alginate, chitosan, hyaluronic acid, hydrocolloids of egg white, and gelatin, have good biocompatibility, biodegradability, and similar structure to the natural extracellular matrix (ECM) and are widely used in tissue engineering scaffolds [7,8,9,10]. Among numerous natural polymer materials, alginate is particularly important in the development and application of tissue engineering scaffolds due to its advantages of non-toxicity, good biocompatibility, non-immunogenicity, biodegradability, low price, abundant source, and easy gelation under mild conditions [11,12,13]. Alginate can form calcium alginate hydrogel through cross-linking of Ca^2+^, which not only inherits all the advantages of alginate but also has coating and protective effects on cells and forms more interconnected 3D network pore structures, which can be used as a transport channel for cell nutrients and metabolic wastes to meet the metabolic activities of cells in the scaffold. Moreover, alginate hydrogel has advantages for bone regeneration because it can be implanted in vivo to fill defective tissue in a minimally invasive manner [14,15]. It has been reported that mesenchymal stem cells (MSCs), human adipose stem cells (hASCs), mouse embryonic osteoblasts (MC3T3-E1), and human osteosarcoma cells (MG-63) can adhere, proliferate, and differentiate on the alginate hydrogel scaffold [11,12,13,14,15] and form the extracellular matrix [16], which provides a research basis for alginate hydrogel as tissue engineering scaffold material [16,17,18,19,20,21].

Polyvinyl alcohol (PVA) is a water-soluble polyhydroxy polymer that can be physically or chemically cross-linked to form the hydrogel as a matrix material for tissue repair and regeneration [22,23,24]. PVA hydrogels have a large number of microporous structures, as well as excellent elastic and compressive properties [25]. Furthermore, PVA has an osmotic equilibrium with the surrounding joint soft tissue. Under stress, PVA hydrogels exhibit a low friction coefficient due to liquid lubrication, which is similar to that of natural articular cartilage [26]. Therefore, PVA hydrogel can be used as a potential substitute material for articular cartilage in tissue engineering [27]. However, the cell affinity of PVA is weak, and it is often compounded with natural materials to construct the composite hydrogels. Therefore, in order to improve the biocompatibility and bioactivity of the PVA hydrogel scaffold, alginate and its hydrogel are incorporated into PVA because of their good bioactivity [15]. Additionally, the Arg-Gly-Asp (RGD) peptide is a tripeptide sequence composed of arginine, glycine, and aspartic acid, which exists in the extracellular matrix of many animals (including humans) and is a common sequence in cell recognition and has a good cell recognition function [28,29,30]. The RGD peptide has a cell adhesion sequence [23] that can mimic cell adhesion proteins and bind to integrins, so it can be used in the synthesis of tissue engineering scaffolds to enhance cell adhesion between cells and tissue engineering scaffolds [31].

Although the construction of composite hydrogel can endow materials with excellent properties [32,33], the weak mechanical strength of composite hydrogel fabricated by PVA and SA limits its application and development in bone tissue engineering. To address this limitation, researchers have added nanomaterials, such as nanoparticles and nanofibers, to such polymer hydrogels to improve their mechanical properties. In addition, nanomaterials, such as nano-montmorillonite, nano-silica, etc., are commonly used to reinforce the PVA matrix [34,35]. However, the nanofillers currently used to reinforce composite hydrogels are generally inorganic, and their processability, biocompatibility, and biodegradability are much lower than organic fillers [36,37]. Notably, cellulose nanocrystals (CNCs) have excellent physicochemical properties such as low density, high aspect ratio, high specific surface area, reproducibility, biodegradability, environmental protection, and low cost [38,39]. As a kind of natural organic nanomaterial, CNCs are rigid crystalline components obtained from the most abundant renewable cellulose on earth by acid, enzyme, mechanical, or ball milling to remove amorphous regions. They can be added to polymers as reinforcing agents, which can effectively enhance their mechanical strength in comparison to other plant cell wall polymers [40]. Hence, owing to the numerous benefits of CNC nanomaterials, they have been found to have extensive applications in enhancing the characteristics of diverse polymer matrix materials.

Taking into account various characteristics of PVA, in this study, PVA and SA were physically blended; ion cross-linking of SA with hydroxyapatite/D-glucono-δ-lactone (HAP/GDL) and physical cross-linking of PVA by a freezing/thawing method constructed interpenetrating network (IPN) composite hydrogels. These composite hydrogels have been widely used in wound dressing and tissue engineering [41,42]. Compared with the chemical cross-linking method, the freeze-thaw technique is a purely physical method, which does not use chemical cross-linking agents to avoid potentially toxic effects on cells, and it has no impact on the non-toxicity, biocompatibility, and biodegradability of polymer hydrogels [43]. Therefore, freeze-thaw technology is a better choice in the preparation of biomedical materials. To further improve the mechanical strength of IPN composite hydrogels, CNCs were added as the reinforcing agent. Since both SA and PVA had weak adhesion to cells, this study explored the grafting of SA with RGD polypeptide sequence after oxidation to enhance cell adhesion to IPN composite hydrogel scaffolds (ADA-RGD/PVA/CNCs). Then, the regulation of the inclusion of PVA on the mechanical properties, morphology and structure, thermal stability, swelling, in vitro biodegradability, and cell compatibility of ADA-RGD/PVA/CNCs was investigated, focusing on the impact of different contents of PVA on the properties of composite hydrogels scaffold materials.

## 2. Results and Discussion

### 2.1. Characterization of Alginate Dialdehyde Grafted RGD Derivatives

**Figure 1:**
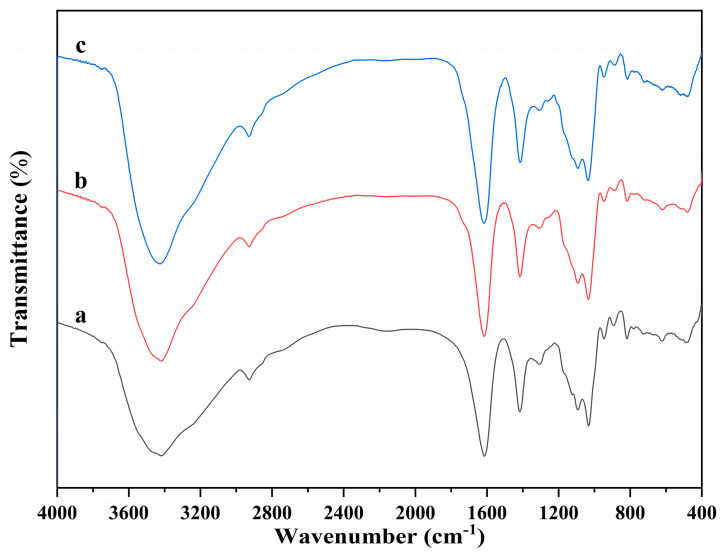
FT-IR spectra of (a) SA, (b) ADA, and (c) ADA-RGD.

The molecular structure of alginate dialdehyde grafted RGD derivatives (ADA-RGD) was monitored by FT-IR and ^1^H NMR. Figure 1 shows the FT-IR spectra of SA, ADA, and ADA-RGD.

Due to the molecular polarity and hydrogen bonding, most of the characteristic absorption peaks of the polymer exhibited a certain degree of overlap and a slight wavenumber shift. It can be observed that the wide peaks of SA appearing in the range of 3600~3000 cm^−1^ were the characteristic peaks of its -OH stretching vibration, while the peaks appearing at 2927, 1615, and 1416 cm^−1^ belonged to the C-H stretching vibration absorption peak and the asymmetric and symmetric stretching vibration characteristic peaks of -COO^−^ on the SA molecular chain, respectively. In addition, C-O and C-C stretching vibration absorption peaks of the SA pyran ring appeared at 1093 and 1032 cm^−1^ [44,45,46]. In contrast, ADA exhibited the FT-IR spectra similar to SA, but no significant stretching vibration characteristic peaks of aldehyde groups could be observed, which may be due to the formation of hemiacetal in ADA under dry conditions [47] or the low number of aldehyde groups generated due to its low oxidation degree. Furthermore, ADA-RGD was a stable product formed by the aldehyde-amine condensation reaction of ADA and RGD and then the reduction of 2-PBC. Its FT-IR spectra were basically consistent with SA and ADA, which may be due to the fact that ADA grafted a small amount of RGD, so its characteristic peaks were not obvious in its FT-IR spectra. In order to further clarify the molecular structure of ADA and ADA-RGD, they were determined by ^1^H NMR technology.

**Figure 2:**
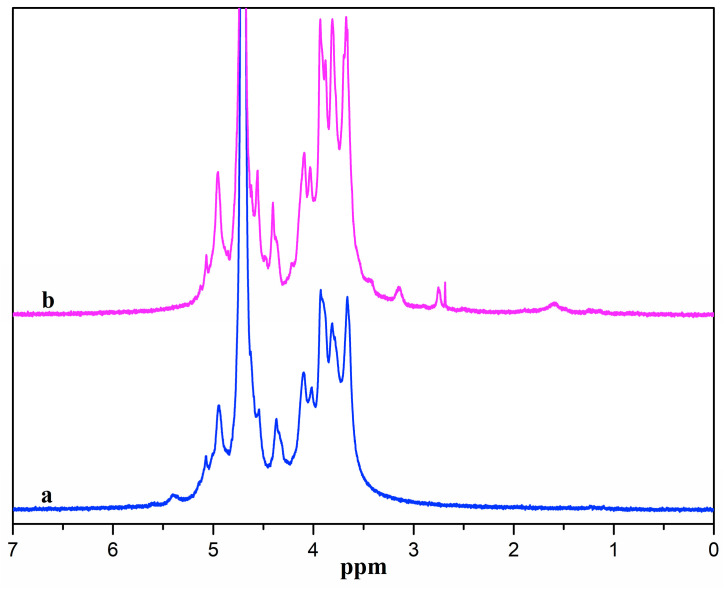
^1^H NMR spectra of (a) ADA and (b) ADA-RGD.

Figure 2 presents the ^1^H NMR spectra of ADA and ADA-RGD. ADA displayed signal peaks in the range of 3.5~5.0 ppm owing to the proton signal peaks in the G and M blocks of alginate [48]. The signal peaks at 5.1 and 5.4 ppm belonged to the proton signal peak of hemiacetal [49], which was due to the formation of hemiacetal between the aldehyde groups and the adjacent hydroxyl groups of ADA, indicating the successful synthesis of ADA. From the ^1^H NMR spectra of ADA-RGD, it was possible to detect the peaks of proton signals originating from the RGD polypeptide chains. The methylene proton peaks at the β and γ positions of arginine were represented by the proton signal peaks ranging from 1.5 to 2.0 ppm [50,51]. Additionally, the methylene proton peak at the α position of arginine was indicated by the signal peak at 3.2 ppm, and the methylene proton signal peak at the α position of aspartate was represented by the signal peak at 2.79 ppm [50,52]. However, the proton peak of glycine did not appear because the position of the proton peak of glycine was near 3.6 ppm and overlapped the proton peaks of alginate [53]. It was also observed that the hemiacetal proton signal peak of ADA-RGD disappeared at 5.4 ppm, which may be the result of 2-PBC reduction of Schiff base or residual aldehyde groups. The above results indicated that RGD was successfully grafted onto the ADA molecular chain to obtain ADA-RGD.

### 2.2. Morphologies of the Composite Hydrogels

**Figure 3:**
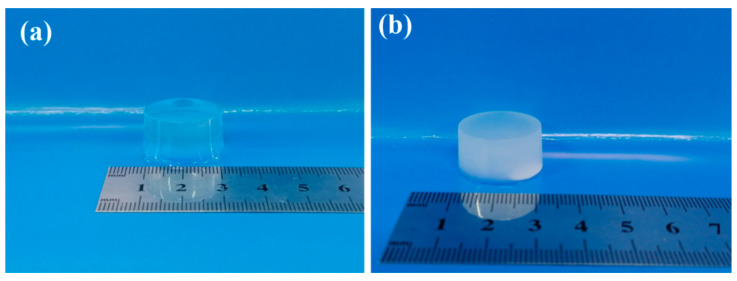
Physical images of pure (**a**) ALG hydrogel and (**b**) ADA-RGD/PVA/CNCs composite hydrogels.

The physical images of the pure ALG hydrogel and the composite hydrogels of ADA-RGD/PVA/CNCs are displayed in Figure 3. The ALG hydrogel was colorless and transparent, while the ADA-RGD/PVA/CNCs composite hydrogels with CNCs and PVA were white and pale blue, which was the result of the addition of nanofillers, CNCs, and the formation of SA/PVA IPN composite hydrogels’ structure.

**Figure 4:**
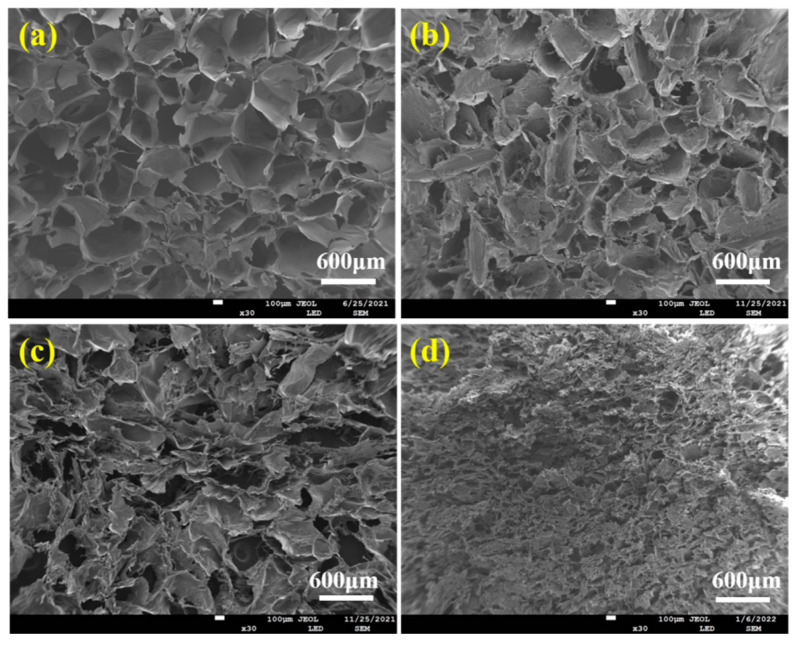
SEM images of (**a**) ALG hydrogel, (**b**) ALG/0.5PVA/0.3%CNCs, (**c**) ALG/PVA/0.3%CNCs, and (**d**) ADA-RGD/PVA/0.3%CNCs composite hydrogels.

Figure 4 displayed the pore structure morphologies of ALG hydrogel, ALG/0.5PVA/0.3%CNCs, ALG/PVA/0.3%CNCs, and ADA-RGD/PVA/0.3%CNCs composite hydrogels. The presence of numerous pore structures in these composite hydrogels allowed for cell proliferation, as well as the transfer of nutrition and metabolic substances [54]. All hydrogel materials left a lot of pore structures in the freeze-drying process as a result of ice crystal sublimation. Table 1 demonstrates that the incorporation of CNCs and PVA into the alginate matrix resulted in a reduction in pore size and a decrease in porosity of the IPN ALG/PVA/CNCs composite hydrogels. This was due to CNCs as a filler and the formation of SA/PVA IPN composite hydrogel structures; the pore wall material increased, thus decreasing the pore structures. However, the pore structures of ADA-RGD/PVA/0.3%CNCs composite hydrogels were irregular, which may be because the support effect of SA on the material was weakened after chemical modification, and some pore walls may collapse after sublimation of ice crystals, so the smaller and more dense pore structures were finally formed.

### 2.3. Characterization of the Composite Hydrogels

**Figure 5:**
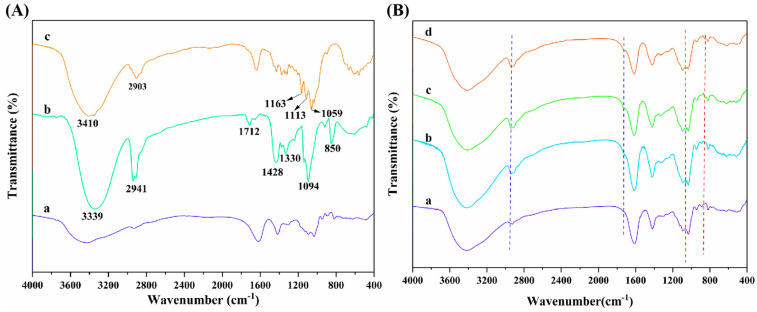
(**A**) FT-IR spectra of (a) SA, (b) PVA, and (c) CNCs; (**B**) FT-IR spectra of (a) ALG hydrogel, (b) ALG/0.5PVA/0.3%CNCs, (c) ALG/PVA/0.3%CNCs and (d) ADA-RGD/PVA/0.3%CNCs composite hydrogels.

Figure 5A displays the FT-IR spectra of SA, PVA, and CNC raw materials. As seen, SA presented the characteristic absorption peaks at 2927, 1615, and 1416 cm^−1^, which were ascribed to the C-H stretching vibration and the asymmetric and symmetric stretching vibration of -COO^−^ on the SA molecular chain, respectively [44,45,46]. Additionally, it can be observed that the strong and wide absorption peak of PVA at 3339 cm^−1^ was mainly caused by the interactions between hydroxyl groups and hydrogen bonding of PVA, which led to the wide absorption band [55]. The absorption peaks at 2941 and 1712 cm^−1^ were the -C-H stretching vibration absorption peak and the C=O stretching vibration characteristic peak, respectively, which are attributed to the fact that PVA molecules contain acetate groups that were not completely hydrolyzed [56]. The absorption peaks at 1428 and 1330 cm^−1^ were caused by C-H bending and rocking vibrations, respectively [57]. In addition, the peaks appearing at 1094 and 850 cm^−1^ were the C-O stretching vibration absorption peaks and C-C stretching vibration absorption peaks of PVA [58]. Furthermore, the absorption peaks of CNCs in the range of 3600~3000 cm^−1^ and 2903 cm^−1^ were the characteristic peaks of -OH stretching vibration and C-H stretching vibration, respectively. The peaks at 1163 and 1113 cm^−1^ were attributed to the C-C bond and C-O-C glucosidoether bond in the pyran ring of CNCs, respectively [59,60].

The interactions and structural changes of components in ADA-RGD/PVA/CNCs composite hydrogels were studied by FT-IR spectroscopy. As can be seen from Figure 5B, ALG hydrogel, ALG/0.5PVA/0.3%CNCs, ALG/PVA/0.3%CNCs, and ADA-RGD/PVA/0.3%CNCs composite hydrogels retained the typical characteristic peaks of SA, PVA, and CNCs. By comparison, it was found that with the addition of PVA and CNCs to the alginate matrix and with the increase of PVA content, the absorption peak of -OH stretching vibration of ALG/PVA/CNCs composite hydrogels in the range of 3600~3000 cm^−1^ revealed a red-shift, which may form the new hydrogen bonds between SA, PVA, and CNCs [54]. With the increase of PVA content in ALG/PVA/CNCs composite hydrogels, the C-H stretching vibration peak at 2926 cm^−1^ presented a blue-shift, which was due to the enhanced intermolecular interactions between PVA and alginate matrix [61]. Meanwhile, the absorption peak of ALG/PVA/CNCs composite hydrogels was observed at 1712 cm^−1^, which was caused by C=O in the unhydrolyzed acetate groups of PVA [56]. Characteristic peaks belonging to CNCs were found in the ALG/PVA/CNCs composite hydrogels, indicating that CNCs were dispersed in the SA/PVA matrix through physical mixing. Moreover, with the increase of PVA content, the intensity of the characteristic PVA absorption peak of ALG/PVA/CNC composite hydrogel at 850 cm^−1^ increased [58]. The results suggested that the IPN structures between PVA, SA, and CNCs were constructed through hydrogen bonding and electrostatic interaction, which had a great influence on the mechanical properties and interfacial interaction of the composite hydrogels.

**Figure 6:**
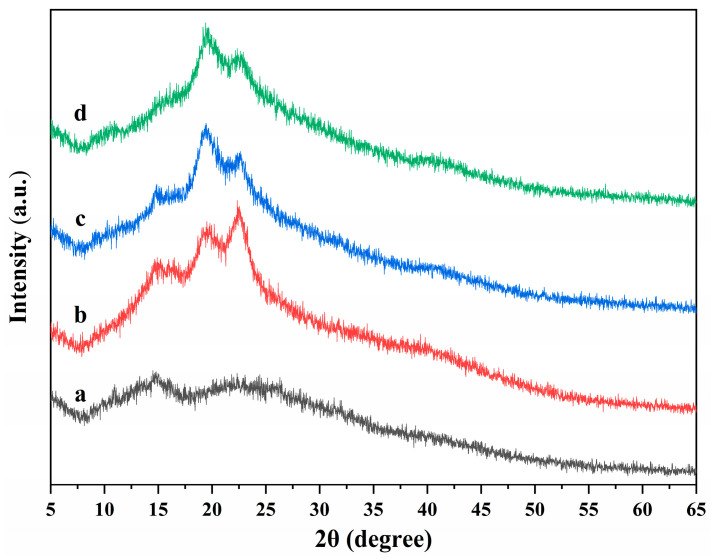
X-ray diffractions of (a) ALG hydrogel, (b) ALG/0.5PVA/0.3%CNCs, (c) ALG/PVA/0.3%CNCs, and (d) ADA-RGD/PVA/0.3%CNCs composite hydrogels.

The X-ray diffractions of ALG hydrogel, ALG/0.5PVA/0.3%CNCs, ALG/PVA/0.3%CNCs, and ADA-RGD/PVA/0.3%CNCs composite hydrogels are displayed in Figure 6. It was observed that the ALG hydrogel showed wide hydrated crystal diffraction peaks at 2θ = 14.8° and 22.7°, indicating that the ALG hydrogel exhibited an amorphous structure [62,63]. With the addition of PVA and CNCs to the SA matrix, ALG/0.5PVA/0.3%CNCs, ALG/PVA/0.3%CNCs, and ADA-RGD/PVA/0.3%CNCs composite hydrogels showed main diffraction peaks at 2θ = 14.8°, 19.8°, and 22.5°. The peak at 2θ = 22.5° was the characteristic diffraction peak of CNCs, and the peak at 2θ = 19.9° was the characteristic diffraction peak of PVA [64]. However, with the increase of PVA content, the peak intensity at 2θ = 22.6° decreased, the peak intensity at 2θ = 19.8° increased, and the peak position shifted to some extent. However, one of the two main diffraction peaks of SA in IPN composite hydrogels was weakened, and the other disappeared. The results showed that the crystal structure of the IPN composite hydrogels formed by CNCs, SA, and PVA changed, which might be the result of an intermolecular hydrogen bond or intramolecular hydrogen bond of SA or ADA-RGD, CNCs, and PVA [65].

**Figure 7:**
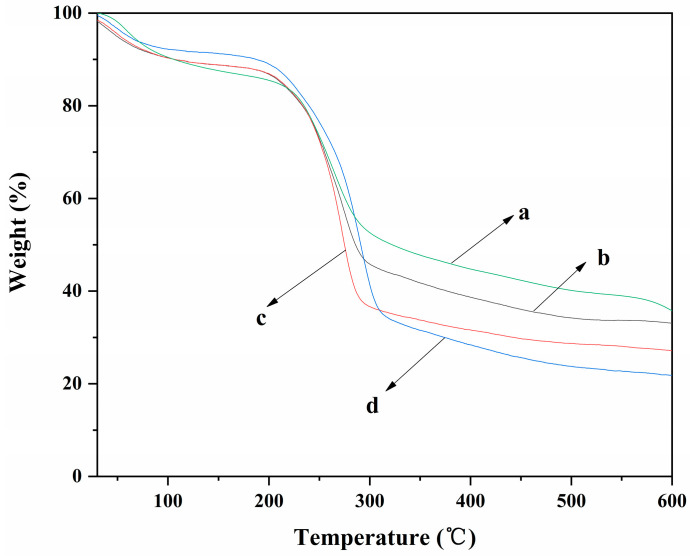
TGA curves of (a) ALG hydrogel, (b) ALG/0.5PVA/0.3%CNCs, (c) ALG/PVA/0.3%CNCs, and (d) ADA-RGD/PVA/0.3%CNCs composite hydrogels.

The TGA curves of ALG hydrogel, ALG/0.5PVA/0.3%CNCs, ALG/PVA/0.3%CNCs, and ADA-RGD/PVA/0.3%CNCs composite hydrogels are shown in Figure 7. It can be observed that each composite hydrogel showed different thermal degradation behavior due to their different components. The weight loss rates of ALG hydrogel, ALG/0.5PVA/0.3%CNCs, ALG/PVA/0.3%CNCs, and ADA-RGD/PVA/0.3%CNCs composite hydrogels were 64.3%, 66.9%, 72.9%, and 78.2%, respectively. The results implied that the addition of PVA and CNCs had a certain effect on the weight loss rate of ALG hydrogel at 600 °C. In the range of 30~105 °C, the removal of physical adsorption water would occur for each composite hydrogel [66]. In the range of 200~315 °C, the polymer in the composite hydrogels was pyrolyzed into some small molecules such as CO_2_, CO, and H_2_O, which led to the rapid decrease of the weight of the composite hydrogels [67]. The pyrolysis temperatures of ALG/0.5PVA/0.3%CNCs, ALG/PVA/0.3%CNCs, and ADA-RGD/PVA/0.3%CNCs composite hydrogels were discovered to be higher than that of ALG hydrogel. Additionally, as the PVA content increased, the pyrolysis temperature of ALG/PVA/0.3%CNCs shifted towards higher temperatures. This shift could be attributed to the formation of an IPN structure through interchain or intramolecular hydrogen bonding and intermolecular entangling of SA, PVA, and CNCs, thereby enhancing the thermal stability of the composite hydrogels [61,68].

**Figure 8:**
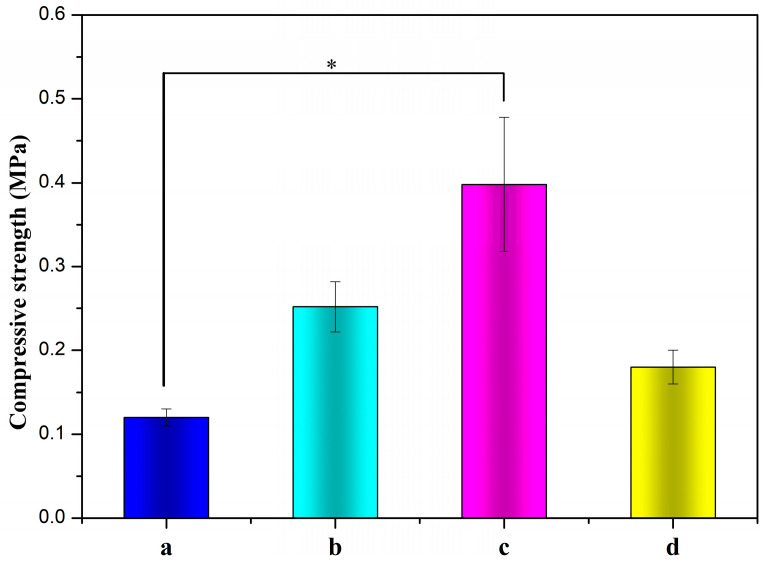
Compressive strengths of (a) ALG hydrogel, (b) ALG/0.5PVA/0.3%CNCs, (c) ALG/PVA/0.3%CNCs, and (d) ADA-RGD/PVA/0.3%CNCs composite hydrogels. * denotes *p* < 0.05, indicating highly significant difference.

The compressive strength of ALG hydrogel, ALG/0.5PVA/0.3%CNCs, ALG/PVA/0.3%CNCs, and ADA-RGD/PVA/0.3%CNCs composite hydrogels are depicted in Figure 8. The compressive strength of IPN ALG/PVA/CNCs composite hydrogels was observed to be greater than that of pure ALG hydrogels, suggesting a significant enhancement in the mechanical properties of ALG hydrogel upon the formation of the IPN structure with PVA and CNCs. By comparing the compressive strength of ALG/0.5PVA/0.3%CNCs and ALG/PVA/0.3%CNCs composite hydrogels, it was observed that the compressive strength increased with the increase of PVA content in the composite hydrogels. This may be because the addition of PVA enhanced the intermolecular entanglement and improved the intermolecular or intramolecular hydrogen bonding [69,70]. The compressive strength of ADA-RGD/PVA/0.3%CNCs composite hydrogels was considerably reduced when compared to ALG/PVA/0.3%CNCs composite hydrogels. This was due to the decrease in the relative molecular weight of SA after chemical modification and the increase in flexibility of the molecular chain due to the ring-opening of the carbon skeleton [71]. In addition, the oxidation process of SA gave rise to the reduction of GG blocks, which reduced the interaction between ADA and Ca^2+^ in ionic cross-linking, thus reducing the compression strength.

### 2.4. In Vitro Swelling and Biodegradation Properties of the Composite Hydrogels

**Figure 9:**
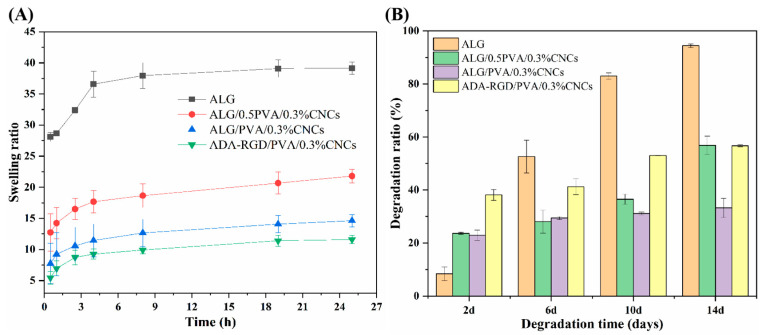
(**A**) Swelling ratio and (**B**) biodegradation ratio of ALG hydrogel, ALG/0.5PVA/0.3%CNCs, ALG/PVA/0.3%CNCs, and ADA-RGD/PVA/0.3%CNCs composite hydrogels.

The swelling curves of ALG hydrogel, ALG/0.5PVA/0.3%CNCs, ALG/PVA/0.3%CNCs, and ADA-RGD/PVA/0.3%CNCs composite hydrogels at different times in PBS solution at 37 °C are shown in Figure 9A. It was observed that the swelling rate of pure ALG hydrogel was the largest, which may be related to the strong hydrophilicity of SA [72]. However, excessive swelling of ALG hydrogel was unfavorable to the mechanical properties and clinical application of the scaffold materials [73]. Compared with ALG hydrogels, the swelling rate of IPN ALG/PVA/CNCs composite hydrogels formed after the addition of PVA and CNCs to the SA matrix was significantly reduced, indicating that the construction of IPN structure and the addition of CNCs could significantly improve the swelling behavior of pure ALG hydrogels and enhance the stability of IPN composite hydrogels in a physiological environment. By comparing the swelling curves of ALG/0.5PVA/0.3%CNCs and ALG/PVA/0.3%CNCs composite hydrogels, it was found that the swelling rate of IPN ALG/PVA/CNCs composite hydrogels further decreased with the increase of PVA content, which may be due to the fact that the addition of PVA led to the formation of the more dense networks, which possessed the smaller apertures and spaces, thus reducing the absorption of PBS solution. In addition, the addition of PVA meant that the percentage of SA and CNCs in the matrix of the composite hydrogels was reduced, resulting in a decrease in the mobility and diffusion of molecular chains, and the overall absorption of PBS by IPN composite hydrogels was also reduced. Additionally, by comparing the swelling curves of ALG/PVA/0.3%CNCs and ADA-RGD/PVA/0.3%CNCs composite hydrogels, it can be found that when the content of PVA and CNCs was constant, the swelling of the composite hydrogels with the use of ADA-RGD was low. Therefore, the swelling property of composite hydrogels could be controlled by adjusting the interpenetrating ratio of components, which laid a foundation for the biological application of alginate composite hydrogel scaffolds [74].

To examine the biodegradation property of the composite hydrogels, ALG hydrogel, ALG/0.5PVA/0.3%CNCs, ALG/PVA/0.3%CNCs, and ADA-RGD/PVA/0.3%CNCs composite hydrogels were immersed in PBS containing 10,000 U/mL lysozyme at 37 °C for in vitro biodegradation experiments. As shown in Figure 9B, ADA-RGD/PVA/0.3%CNCs showed a higher degradation rate at different times than other IPN composite hydrogels, which may be due to the low molecular weight and oxidized uronic acid residues of ADA-RGD in the physiological environment, which made it more prone to alkali elimination reaction. The alkali elimination reaction promoted the degradation of ADA-RGD [75,76], so the chemical modification of SA improved the biodegradation rate of IPN composite hydrogels. By comparing ALG hydrogel, ALG/0.5PVA/0.3%CNCs, and ALG/PVA/0.3%CNCs composite hydrogels, it was found that the addition of CNCs and the increase of PVA content reduced the degradation rate of IPN ALG/PVA/CNCs composite hydrogels. This may be ascribed to the intramolecular or intermolecular hydrogen bonding between SA, CNCs, and PVA and the formation of a more dense cross-linking network with the increase of PVA content to hinder the erosion of lysozyme and water [61,65]. Therefore, the addition of PVA could control the biodegradation rate of IPN ALG/PVA/CNCs composite hydrogels. The results indicated that the degradation rate of ALG/PVA/0.3%CNCs constructed by IPN technology was controllable, which was of great significance for the biological application of IPN composite hydrogels. 

### 2.5. In Vitro Cytocompatibility of the Composite Hydrogels

**Figure 10:**
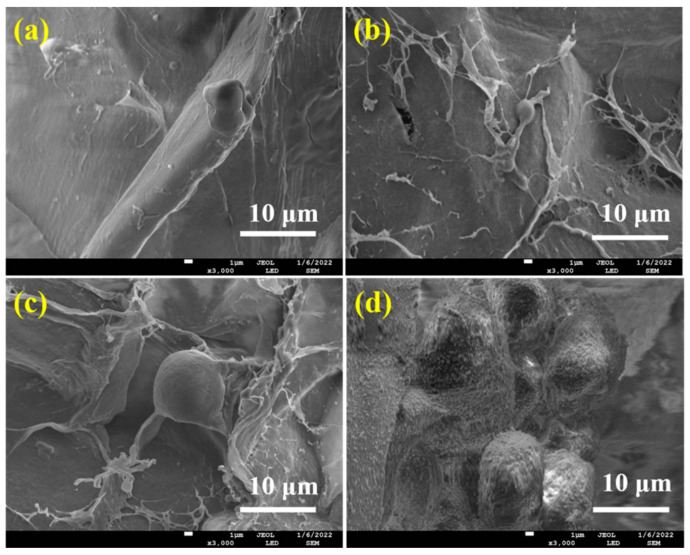
SEM images of MC3T3-E1 cells cultured on (**a**) ALG hydrogel, (**b**) ALG/0.5PVA/0.3%CNCs, (**c**) ALG/PVA/0.3%CNCs, and (**d**) ADA-RGD/PVA/0.3%CNCs composite hydrogels for 2 d.

The cell attachment, growth, and differentiation of ALG hydrogel, ALG/0.5PVA/0.3%CNCs, ALG/PVA/0.3%CNCs, and ADA-RGD/PVA/0.3%CNCs composite hydrogels were evaluated with the mouse MC3T3-E1 cells. The SEM images of MC3T3-E1 cells cultured on these IPN composite hydrogels for 2 d are shown in Figure 10. Cell adhesion was observed on all IPN composite hydrogels, but only a small amount of cell adhesion was observed on ALG hydrogel, ALG/0.5PVA/0.3%CNCs, and ALG/PVA/0.3%CNCs composite hydrogels. As SA and PVA lacked cell recognition sites, their cell affinity was weak. However, MC3T3-E1 cells displayed good adhesion on the surface of ADA-RGD/PVA/0.3%CNCs composite hydrogels and formed cell communities on the surface of the material, indicating that RGD grafting could improve cell adhesion on the surface of the IPN composite hydrogels [30,54].

**Figure 11:**
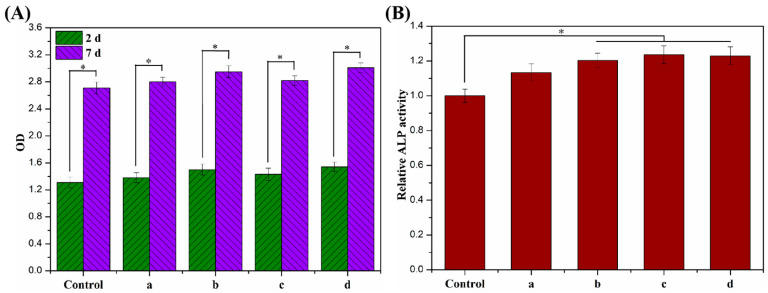
(**A**) Cell proliferation viability and (**B**) cell differentiation of MC3T3-E1 cells cultured on (a) ALG hydrogel, (b) ALG/0.5PVA/0.3%CNCs, (c) ALG/PVA/0.3%CNCs, and (d) ADA-RGD/PVA/0.3%CNCs composite hydrogels for 2 d and 7 d. * denotes *p* < 0.05, indicating highly significant difference.

The proliferative activity of MC3T3-E1 cells cultured on ALG hydrogel, ALG/0.5PVA/0.3%CNCs, ALG/PVA/0.3%CNCs, and ADA-RGD/PVA/0.3%CNCs composite hydrogels for 2 d and 7 d were detected by a CCK-8 kit, and the results are shown in Figure 11A. After 2 d culture, the proliferation activity of MC3T3-E1 cells on ALG hydrogel, ALG/0.5PVA/0.3%CNCs, ALG/PVA/0.3%CNCs, and ADA-RGD/PVA/0.3%CNCs composite hydrogels was higher than that of the control group, indicating that the cells showed better proliferation activity on these composite hydrogels, which was due to the porous structure of IPN composite hydrogels which provided a three-dimensional space for the cell proliferation [65]. After 7 days of culture, cells showed an obvious proliferation effect on all the IPN composite hydrogels, among which the proliferation activity of ALG/0.5PVA/0.3%CNCs and ADA-RGD/PVA/0.3%CNCs composite hydrogels was significantly higher than that of the control group and other groups. This was closely related to the well-developed pore structure and RGD-grafted activity of the composite hydrogels [61].

In addition, the cell differentiation of MC3T3-E1 cells cultured on ALG hydrogel, ALG/0.5PVA/0.3%CNCs, ALG/PVA/0.3%CNCs, and ADA-RGD/PVA/0.3%CNCs composite hydrogels for 7 d was determined by an alkaline phosphatase (ALP) kit (Biyuntian Biotechnology Co., Ltd., Shanghai, China) [54]. As shown in Figure 11B, the relative ALP activity of cells on each IPN composite hydrogel was higher than that of the control group, indicating that the porous IPN composite hydrogel helped to promote the differentiation of the MC3T3-E1 cells. Compared with ALG hydrogel, ALG/0.5PVA/0.3%CNCs, ALG/PVA/0.3%CNCs, and ADA-RGD/PVA/0.3%CNCs composite hydrogels exhibited more significant relative ALP activity. In contrast, the relative ALP activity of ALG/PVA/0.3%CNCs and ADA-RGD/PVA/0.3%CNCs composite hydrogels was significantly higher than that of the control group, which may indicate that PVA and CNCs had good biocompatibility, which was conducive to cell proliferation and differentiation. Moreover, the excellent biological activity of ADA-RGD greatly promoted the osteogenic differentiation of MC3T3-E1 cells [73].

## 3. Materials and Methods

### 3.1. Materials

Sodium alginate (SA, biochemical grade, MW = 438,000), polyvinyl alcohol (PVA, 98%, alcoholysis degree: 87.0–89.0 mol%), hydroxyapatite (HAP, 98%), D-glucono-δ-lactone (GDL, 99%), 2-methylpyridine borane complex (2-PBC, 95%), and Arg-Gly-Asp (RGD, 97%) were purchased from Aladdin Biochemical Technology Co., Ltd. (Shanghai, China). Alginate dialdehyde (ADA) with theoretical oxidation degrees of 10% was synthesized by specific oxidation of sodium periodate [67]. Cellulose nanocrystals (CNCs) were acquired by sulfuric acid hydrolysis of microcrystalline cellulose based on our previous report [77]. Other biochemical reagents, including MC3T3-E1 cells, culture medium, antibiotics, and assay kits, were purchased from Gibco, Thermo Fisher Scientific Co., Ltd. (Waltham, MA, USA).

### 3.2. Preparation and Structural Characterization of ADA-RGD

**Figure 12:**
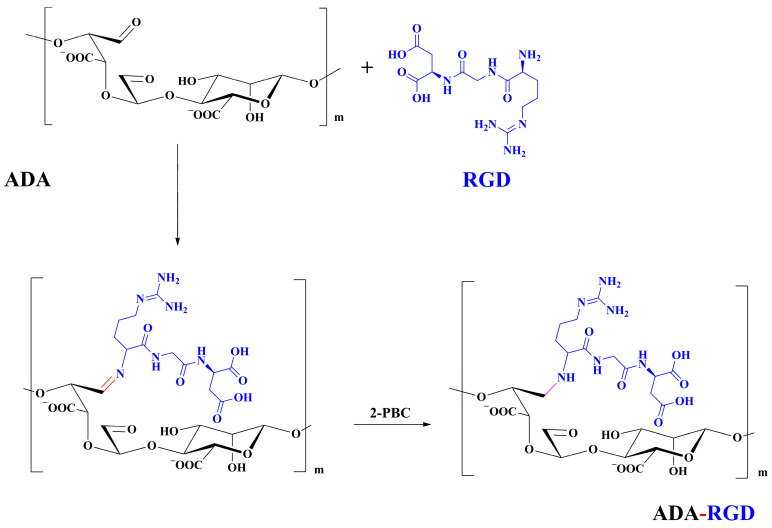
Synthesis route of ADA-RGD.

The synthesis route of ADA-RGD is illustrated in Figure 12. Briefly, 1.00 g of ADA was dissolved in 50 mL ultra-pure water under the action of magnetic stirring, and then 40 mg of RGD sequence polypeptide (the amount of polypeptide was 115.5 μmol/g ADA) was added to the ADA solution. Afterward, the Schiff base reaction was induced by stirring at room temperature. After 24 h, 0.11 g (20 mM) of 2-PBC was added to the reaction solution, stirring for 2 h, reducing the -C=N- bond to a stable -C-N- bond and reducing the unreacted aldehyde groups. Finally, the reaction solution was poured into a dialysis bag (MWCO 8000 Da) and dialyzed against ultra-pure water for 7 d. Then, the purified solution was lyophilized to obtain ADA-RGD with a yield of 73.3%.

Subsequently, the FT-IR and ^1^H NMR techniques were employed to analyze the molecular composition of ADA-RGD. The Nicolet-6700 (ThermoScientific, Waltham, MA, USA) was used to record the FT-IR spectra of the sample. The spectra were obtained using KBr pellets and covered a wavenumber range of 4000 to 400 cm^−1^. The recording involved 64 scans with a spectral resolution of 2.0 cm^−1^. The ULTRASHIELD 400 PLUS spectrometer (Bruker, Fällanden, Switzerland) was used to record the ^1^H NMR spectra at 25 °C. The spectrometer operated at 400 MHz, and deuterated water (D_2_O) was used as the solvent. Tetramethylsilane (TMS) served as the internal standard, and the sample had a concentration in the range of 8.0~10.0 mg/mL.

### 3.3. Fabrication of ADA-RGD/PVA/CNCs Composite Hydrogels

The fabrication of ADA-RGD/PVA/CNCs composite hydrogels was based on our previous method with some modifications [65]. In detail, 137 mg of HAP was accurately weighed and dissolved in 100 mL 0.3% (*w*/*v*) CNCs solution, and the uniformly dispersed HAP/CNCs mixed solution was obtained by mechanical stirring under the action of ultrasound. After 1.50 g of PVA was added to the mixed solution, it was mechanically stirred at 90 °C for 2 h to dissolve the PVA. Afterward, 1.50 g of ADA-RGD was added, and then the mixed solution of ADA-RGD/PVA/CNCs was formed by mechanical stirring for 2 h. Subsequently, a 30 mL sterile syringe was used to inject 15 mL of the ADA-RGD/PVA/CNCs mixed solution into a 50 mL beaker. Furthermore, 109 mg of GDL was introduced to facilitate in-situ ion cross-linking of ADA-RGD under magnetic stirring. Four min later, approximately 5 mL of viscous solution was promptly transferred to a 12-well tissue culture plate (TCP) via the sterile syringe. This TCP was then sealed with plastic wrap to prevent the evaporation of the liquid. After undergoing cross-linking at a temperature of 4 °C for a duration of 4 h, the ADA-RGD/PVA/CNCs mixture was frozen at −24 °C for 12 h and then thawed at 4 °C for 12 h. After four cycles of freezing and thawing, the dehydrated IPN composite hydrogels of ADA-RGD/PVA/0.3%CNCs composite hydrogels were prepared by freeze-drying technology. For comparison, ALG/PVA/0.3%CNCs and ALG/PVA/0.3%CNCs composite hydrogels with different SA/PVA mass ratios of 1:0.5 and 1:1 were respectively fabricated using the aforementioned technique. Moreover, ALG hydrogel was also prepared as the reference.

### 3.4. Characterization of the Composite Hydrogels

A JSM-7100F scanning electron microscope (JEOL, Tokyo, Japan) and an Auto Pore IV 9500 Mercury porosimeter (Beijing, China) were used to examine the pore structure of the cross-section of the composite hydrogels after freeze-drying.

The composite hydrogels’ functional groups and inter-component interactions were analyzed using a Nicolet 6700 FT-IR spectroscopy instrument from Thermo Fisher Scientific (Waltham, MA, USA). The test samples were prepared using the KBr tablet technique. The test parameters were implemented with a resolution of 4 cm^−1^, 32 scans, and a scanning wavenumber range from 4000 to 400 cm^−1^.

The Rigaku UItima-IV X-ray diffraction (Tokyo, Japan) was utilized to conduct XRD examination on different composite hydrogel powders. X-ray diffraction patterns of the samples were obtained at room temperature using Cu-Kα radiation as the X-ray source. The test voltage and current were set at 40 kV and 40 mA, respectively. The scanning speed was 0.03°/s, covering a 2θ range from 5° to 65°.

The thermal stability of different composite hydrogels was analyzed using a TA Q600 thermogravimetric analyzer (New Castle, DE, USA) under a N_2_ atmosphere. The detection was conducted at a heating rate of 10 K/min within a temperature range of 25–500 °C.

The freeze-dried composite hydrogels underwent compression tests utilizing a WDW-1 computerized electronic universal tensile testing machine (Yinuo Century Testing Instrument Co., Ltd., Jinan, China). The freeze-dried composite hydrogels were positioned beneath the compressive disk and subjected to perpendicular longitudinal compression relative to the sample’s surface. After reaching a strain of over 60%, the sample was halted with a compression rate of 5 mm/min. On the stress-strain curves recorded by the instrument, the highest linear point was the compressive strength of the sample. Each sample was measured in parallel 5 times to take the average value.

### 3.5. In Vitro Swelling and Biodegradation Behaviors

The swelling behavior of the composite hydrogels was investigated using the gravimetric technique. W_Dry_ was the recorded weight of the samples after freeze-drying. Once the designated time period had elapsed, the samples were removed from the PBS solution (pH 7.4) at a temperature of 37 °C. The wet samples were weighed as W_Wet_ after removing any extra PBS solution from the samples’ surface using filter paper. The swelling ratio of each sample was calculated in every interval using Equation (1) [54]. For every sample, three measurements were taken in parallel, and the average value was calculated.

(1) $$S\mathrm{welling}\;\mathrm{ratio}=\frac{W_{\mathrm{Wet}}-W_{\mathrm{Dry}\;}}{W_{\mathrm{Dry}}}$$

The weight change of ALG/SS/CNCs composite hydrogels was measured to evaluate their biodegradabilities after being incubated in PBS buffer with 10,000 U/mL lysozyme for 14 d. The sample was freeze-dried, weighed, and recorded as W_Initial_. Then, it was immersed in pH 7.4 PBS at 37 °C for durations of 2, 6, 10, and 14 d. The sample was withdrawn at regular intervals, cleansed multiple times using ultrapure water, and then freeze-dried to measure its weight as W_Dry_. Consequently, three parallel experiments were conducted for each sample to calculate the biodegradation ratio using Equation (2) [61].

(2) $$\mathrm{Biodegradation}\;\mathrm{ratio}=\frac{W_{\mathrm{Initial}\;}-W_{\mathrm{Dry}\;}\;}{W_{\mathrm{Initial}\;}}\times 100\%$$

### 3.6. Cytocompatibility of the Composite Hydrogels

To test the cytocompatibility of the composite hydrogels, a selected population of mouse osteoblasts MC3T3-E1 was cultured in a medium containing 90% DMEM, 10% fetal bovine serum, 100 U/mL penicillin, and 100 μg/mL streptomycin, which could imitate osteoblastic growth. After being exposed to cobalt 60 radiation at a strength of 8 kGy, the tested samples with 14 mm in diameter and 3 mm in height were subsequently transferred to the 24-well tissue culture plates. Afterward, MC3T3-E1 cells were placed onto the composite hydrogels with a density of 5 × 10^4^ per well, whereas the identical cells were seeded on the tissue culture plate without any material as the control group. Throughout this period, the culture medium was enhanced in order to uphold a combined capacity of 500 μL per well. The cells were incubated at a temperature of 37 °C in a culture chamber with a mixture of 5% carbon dioxide, 95% oxygen, and 100% humidity, and the culture medium was refreshed every 48 h.

SEM was used to examine cell adhesion and spreading on the composite hydrogels. The composite hydrogels were cross-linked with glutaraldehyde, dehydrated in a graded ethanol series, and then freeze-dried after 2 d of incubation. Following this, the cell growth on the composite hydrogels was assessed using the Cell counting Kit-8 (CCK-8) assay as described in our previous study [78]. After a time interval of 2 d and 7 d, 50 μL of CCK-8 solution was introduced to 500 μL of medium in every well of 24-well culture plates. The mixture was then incubated at a temperature of 37 °C for a duration of 4 h within an incubator. In the end, a volume of 100 μL of solution was moved to each well in a 96-well plate. Afterward, the optical density (OD) value at a wavelength of 450 nm was measured using the Bio-rad X-mark microplate readers (Hercules, CA, USA). To analyze the process of osteogenic differentiation, the cells on the composite hydrogels were examined using the alkaline phosphatase (ALP) kit. To enhance cell differentiation, the culture medium was supplemented with a mixture of 0.8 mmol/L β-glycerophosphate and 0.2 mol/L dexamethasone. After incubating for 7 d, the cells were washed with PBS, disrupted in an ice bath containing 0.2% (*w*/*w*) Triton X-100, and then centrifuged at 12,000 r/min for 5 min at 4 °C. After that, a total of 50 μL of the centrifugation supernatant was gathered and combined with an equal amount of ALP staining reagent. Following a 30-min period of incubation at 37 °C, the optical density (OD) value of the cells on the composite hydrogels at 405 nm wavelength was also examined by the X-mark microplate reader to determine the relative activity of ALP.

### 3.7. Statistical Analysis

The data were analyzed by data statistics software and processed by Excel. The results were expressed as mean ± standard deviation, and the comparison of variables was analyzed by one-way variance; *p* < 0.05 indicated that the difference was statistically significant.

## 4. Conclusions

In summary, in order to improve the mechanical properties and biological activity of alginate hydrogel, IPN ALG/PVA/CNCs composite hydrogels were fabricated by constructing the IPN hydrogel structure of SA and PVA using CNCs as the reinforcement agent. The resultant IPN ALG/PVA/CNCs composite hydrogels with regular morphology and porous structure were successfully prepared under the interaction of HAP/GDL internal ion cross-linking and freeze-thaw physical cross-linking. The pore size and porosity of the IPN ALG/PVA/CNCs composite hydrogels could be regulated by adjusting PVA content and the addition of CNCs. CNCs with high specific surface area could form a dense interface with the polymer matrix. By increasing the PVA content, the number of physical cross-linking points in PVA increased, resulting in greater stress support for the IPN composite hydrogels of ALG/PVA/CNCs and consequently improving their mechanical characteristics. The creation of the IPN ALG/PVA/CNCs composite hydrogels’ physical cross-linking network through intramolecular or intermolecular hydrogen bonding led to improved thermal resistance and reduced swelling and biodegradation rate. Conversely, the ADA-RGD/PVA/CNCs IPN composite hydrogels exhibited a quicker degradation rate, attributed to the elimination of ADA-RGD by alkali. Furthermore, ALG/0.5PVA/0.3%CNCs and ADA-RGD/PVA/0.3%CNCs composite hydrogels showed better proliferative activity, while ALG/PVA/0.3%CNCs and ADA-RGD/PVA/0.3%CNCs composite hydrogels displayed obvious proliferation effects. To note, PVA and CNCs with good biocompatibility were conducive to cell proliferation and differentiation, and the excellent biological activity of ADA-RGD greatly promoted cell adhesion and osteogenic differentiation for the IPN composite hydrogels, which favor developing strategies to deliver cell therapies for wound healing and tissue engineering.

## Figures and Tables

**Table 1:** Porosity of ALG hydrogel and IPN ALG/PVA/CNCs composite hydrogels.

Sample	Porosity (%)	Mean Pore Size (μm)
ALG hydrogel	91.7 ± 4.1	430 ± 40
ALG/0.5PVA/0.3%CNCs	91.0 ± 0.7	310 ± 34
ALG/PVA/0.3%CNCs	86.4 ± 1.8	280 ± 29
ADA-RGD/PVA/0.3%CNCs	85.6 ± 4.8	110 ± 22

## Data Availability

The data presented in this study are available on request from the corresponding author.

## Sample Availability

Samples of the compounds are available from the authors.
